# Comparison between Liquid and Tablet Formulations in the Treatment of Congenital Hypothyroidism up to 3 Years of Age: The First Italian Study

**DOI:** 10.3390/children11091136

**Published:** 2024-09-19

**Authors:** Rita Ortolano, Erika Cantarelli, Federico Baronio, Valentina Assirelli, Egidio Candela, Carla Mastrangelo, Sofia Vissani, Randa S. Alqaisi, Marcello Lanari, Alessandra Cassio

**Affiliations:** 1 Pediatric Unit, IRCCS Azienda Ospedaliero-Universitaria di Bologna, 40138 Bologna, Italy; rita.ortolano@aosp.bo.it (R.O.); federico.baronio@aosp.bo.it (F.B.); valentina.assirelli3@unibo.it (V.A.); sofia.vissani2@unibo.it (S.V.); marcello.lanari@unibo.it (M.L.); alessandra.cassio@unibo.it (A.C.); 2 Specialty School of Pediatrics, Alma Mater Studiorum, University of Bologna, 40126 Bologna, Italy; erika.cantarelli@studio.unibo.it; 3 Department of Medical and Surgical Sciences, Alma Mater Studiorum, University of Bologna, 40126 Bologna, Italy; 4 Faculty of Medicine, Alma Mater Studiorum, University of Bologna, 40126 Bologna, Italy; carla.mastrangelo@studio.unibo.it; 5 Pediatric and Neonatology Department, Faculty of Medicine and Surgery, Mutah University, Alkarak 61710, Jordan; randasq@mutah.edu.jo

**Keywords:** congenital hypothyroidism, levothyroxine formulation, levothyroxine liquid formulation, newborn screening, neurodevelopmental outcome, levothyroxine efficacy

## Abstract

**Background/Objectives**: Levothyroxine (L-T4) is available for use in congenital hypothyroidism (CH) in three formulations: tablets, drops, and oral solution. This study aims to compare the efficacy and safety of all three L-T4 formulations. **Methods**: We enrolled 63 children born between January 2019 and April 2023 in the Emilia-Romagna Region (Italy) and diagnosed with CH by newborn screening. They were divided according to the L-T4 formulation used: drops (Group D), oral solution (Group S), and tablets (Group T). Clinical and laboratory data were collected up to 3 years after the start of replacement therapy. **Results**: Serum-free thyroxine (sFT4) and thyroid stimulating hormone (sTSH) normalization occurred within the first month of treatment in most patients of all groups. No negative effects on growth and cognitive development were observed. At 7–15 days we found higher median sTSH levels (*p* = 0.031) and a greater percentage of patients with sTSH > 5 µU/mL (*p* = 0.011) in Group S than in Group T, but comparable sFT4 levels. At 12 months, a greater percentage of patients of Group D showed sFT4 values below the normal range than Group S (*p* = 0.011) and Group T (*p* = 0.038); **Conclusions**: Overall, our study reported an equal efficacy of the L-T4 oral solution compared to drops and tablets in CH treatment. A larger series of patients and a long-term follow-up are needed.

## 1. Introduction

Congenital hypothyroidism (CH) is the primary preventable factor leading to neurodevelopmental disability [1]. The incidence of primary CH is around 1/2000–3000 live births [1]. In recent years, there has been an increasing trend in the incidence of CH, mostly attributed to the use of lower Thyroid-Stimulating Hormone (TSH) cut-off values in newborn screening programs [2,3]. Timely identification and intervention are essential to attain a level of psychomotor and neurocognitive development comparable to that of a healthy population.

Levothyroxine (L-T4) is available in three distinct formulations: tablets, which were the first form introduced; drops, a liquid formulation that has been utilized in Italy since 2009; and lastly, the oral solution, which received approval for CH therapy in Italy in December 2018.

Prior research conducted a comparison between tablets and drops. The last one exhibited a comparable result, providing regular growth and neurodevelopment.

During the first month of medication, there was a significant major suppression in serum TSH (sTSH) levels, suggesting a larger likelihood of overtreatment with drops compared to tablets [4,5,6]. The theory proposed to account for this phenomenon was the enhanced bioavailability and absorption of the liquid formulation in the drop form and the loss of part of the active ingredient in the crushing process necessary for administering tablets in children [5]. This data is relevant in the initial stages of pathology care, indicating the need for lower dosages and more frequent monitoring [6]. Indeed, persistent overtreatment has been correlated by Bongers-Schokking et al. to both a long-term cognitive deficiency and an increased risk of permanent attention deficit hyperactivity disorder [7,8]. The inclusion of ethanol as an excipient in liquid formulations in the form of drops and its possible negative consequences have been extensively debated in recentyears. The maximum dose based on body weight (15 mg/kg/day) corresponds to an ethanol amount of 36.45 mg/kg, which is four times lower than the danger threshold of 150 mg/kg recommended by the American Academy of Pediatrics [9,10]. However, the long-term consequences of chronic use of ethanol in pediatric age are yet unclear. This excipient is not present in the formulation of the oral solution.

Due to the recent introduction of the L-T4 oral solution, there is a lack of studies on a wide scale that have compared the clinical outcomes of patients treated with the L-T4 oral solution to those treated with the other two formulations.

Our study aims to assess the effectiveness and safety of the novel oral solution formulation of L-T4 in comparison to the liquid drop formulation and the tablet formulation.

## 2. Materials and Methods

### 2.1. Patients and Study Design

**Figure 1:**
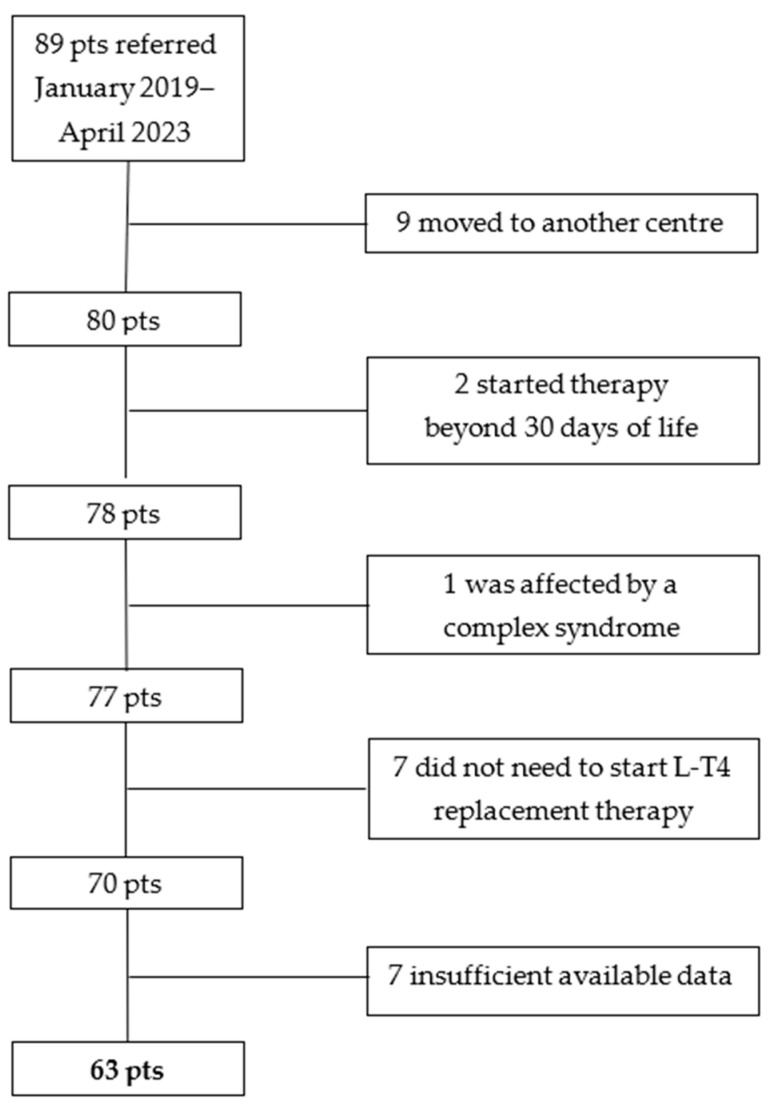
The selection process of patients identified at neonatal screening for suspected CH. Pts.: patients.

A single-center retrospective study was conducted on a cohort of children of both genders born between January 2019 and April 2023 in the Emilia-Romagna Region (Italy). These children were diagnosed with CH through newborn screening (NBS) performed at our regional screening center (IRCCS University Hospital, Bologna, Italy). The diagnostic confirmation and subsequent monitoring of kids with CH were conducted at the Unit of Pediatric Endocrinology of the IRCCS. The research included individuals with confirmed congenital hypothyroidism who began L-T4 replacement medication within the first 30 days after birth. Infants who had documented chromosomal abnormalities and complicated syndromes or were moved to another medical facility before completing at least 12 months of follow-up were excluded. The procedure of selecting patients is depicted in Figure 1.

Overall, 63 patients were enrolled in the study, and they were divided according to the kind of L-T4 formulation used: 22 patients were treated with drop formulation (Group D), 16 with oral solution (Group S), and 25 with tablets (Group T). The formulation was chosen based on clinical practice, involving healthcare provider preferences and familiarity with each pharmaceutical formulation and immediate availability of the formulations, and took into account preferences and features of the family, such as compliance, accuracy in dosing therapy (drops can be more easily overdosed due to excess drops falling, while part of the active ingredient can be missed in the crushing procedure of solid formulation), and family daily habits (solid formulation requires a more stringent observance of the fasting time). The starting L-T4 dosage was defined according to European CH Guidelines [1] and adjusted as needed to reach normal serum TSH and free thyroxine (FT4) levels for age.

### 2.2. Methods

The TSH cut-off value on dried blood spot (DBS) was consistently set at 9 mUI/L during the whole trial period. Upon confirming the diagnosis, the following procedures were conducted: assessment of family history and pre-perinatal history, clinical examination, evaluation of serum TSH, FT3, and FT4 (sFT4) levels, and thyroid ultrasound (US) to determine the existence of the thyroid gland in its normal cervical position. In cases where thyroid tissue was not seen in the cervical region, Technetium-99m (^99^TC) scintigraphy was performed to determine the presence of an ectopic thyroid gland or athyreosis.

Measurement of sTSH and sFT4 was performed using the electrochemiluminescence technique (ECLIA) with the commercial kit Roche Diagnostics (Mannheim, Germany) with a coefficient of variation (CV) of 5%. Thyroid ultrasound was conducted using a real-time probe at a frequency of 12 MHz. It was performed on the patient in a supine position, and the neck was hyperextended. Morphology values and thyroid volume were defined by comparing measurements obtained from those of a healthy population from the same clinical center and data from the literature [11].

Scintigraphy of the thyroid gland was performed. Patients underwent sonographic evaluation of the thyroid gland after intravenous administration of ^99m^Tc-natrium (sodium) pertechnetate (^99m^TcO_4_^−^) as recommended by practice guidelines of the European Association of Nuclear Medicine (EANM) and the Society of Nuclear Medicine and Molecular Imaging (SNMMI) thyroid [12]. ^99^TC-pertechnetate is a pharmacologic mimic of iodine, which is concentrated within the thyroid cells by the sodium-iodide symporter (NIS) activity; however, it is not organized, and therefore washout from thyroid cells occurs after 30 min of radiotracer injection. Scintigraphy enables the determination of the ^99m^Tc-pertechnetate uptake; its distribution is a very accurate method for the diagnosis and quantification of thyroid autonomy and the detection of ectopic thyroid tissue.

We considered thyroid dysgenesis: athyreosis, ectopic thyroid, and severe thyroid hypoplasia. The latter was arbitrarily considered based on a US antero-posterior diameter of 4 mm or less. This choice was supported by our clinical experience and previous report of a major incidence of permanent CH in smaller thyroid glands [13]. This is a significant factor to take into consideration when evaluating homogeneity between groups.

During the initial 36-month period of observation (median 27 months, range 12–36 months), we gathered information about clinical characteristics (weight, height, head circumference, signs and symptoms of over- or under-treatment), blood tests (sTSH and sFT4), and L-T4 dose (µg/kg/day). We examined seven main time points from the initiation of L-T4 replacement therapy: 7–15 days, 1 month, 3 months, 6 months, 12 months, 24 months, and 36 months. Furthermore, we assessed the frequency of treatment overdose (number of sTSH < 0.5 µU/mL/total number of sTSH examinations) and underdose (number of sTSH > 5 µU/mL/total number of sTSH examinations) during the first, second, and third years of follow-up for each patient.

A professional pediatric psychologist assessed psychomotor and neurocognitive development at 12 months using Griffiths Mental Development Scales (GMDS) and at 36 months of age using Wechsler Preschool and Primary Scale of Intelligence-III (WPPSI-III). The Global Developmental Quotient (GDQ) is evaluated by GDMS, along with six sub-scales: A: locomotor (gross motor skills), B: personal-social (skills in daily living activities, level of independence, and interaction with other children); C: hearing and language; D: eye and hand coordination (fine motor skills, manual proficiency, and visual monitoring ability); E: performance (ability to reason through tasks, including speed of working and precision). The normal DQ is as falling between 85 and 115, with a mean normative value of 100. Mild development delay is classified as a DQ between 68 and 84, whereas severe developmental delay is defined as a DQ of 68 or below [14,15].

The WPPSI-III assessment consists of 14 subtests, including 7 verbal tests, 5 performance tests, and 2 tests measuring speed of working. It provides scores for global developmental quotient (DQ), verbal DQ, and performance DQ. Normally developing kids present a mean score of each DQ of 100, with a standard deviation (SD) of 15 [16].

### 2.3. Statistical Analysis

Population characteristics are represented as absolute frequencies and percentages for categorical data and as mean ± SD or median and range (lowest and highest value for each data point) for continuous data. A non-parametric statistical analysis was performed, considering the limited sample size. The Kruskal–Wallis test was used to compare the continuous data of the three groups. The analysis of categorical data has been conducted using the χ^2^ or Fisher exact test, as deemed suitable. Statistically significant results were defined as having *p* values less than 0.05. The statistical analysis was conducted using JASP software version 0.17.2.1 (Amsterdam, The Netherlands).

### 2.4. Ethical Issue

The study adhered to the principles of the Declaration of Helsinki and obtained clearance from the Ethics Committee of the Hospital (Prot. n. 204/2024 n°12/2024/Oss/AOUBo) with nulla osta for no profit study.

## 3. Results

### 3.1. Clinical and Hormonal Characteristics of the Patients at Diagnosis

Table 1 shows patients’ characteristics at diagnosis divided into three groups according to the L-T4 formulation used.

Among the patients in our cohort, the most common CH etiology was thyroid dysgenesis, which was observed in 63.5% (40/63) of the patients. Specifically, 67.5% (27/40) of the patients had thyroid ectopia, 25% (10/40) had thyroid agenesis, and 7.5% (3/40) had severe hypoplasia. In situ thyroid was observed in 57.5% (23/40) of patients. Among these, 13 patients were later determined to have transient CH during the follow-up period (2 in Group D, 3 in Group S, 8 in Group T), 4 patients were diagnosed with permanent CH (2 in Group D, 2 in Group T), and 6 patients have not yet had re-evaluation for diagnosis. The prevalence of thyroid dysgenesis, transient in situ thyroid, and permanent in situ thyroid was comparable across the three groups.

Table 2 displays the TSH levels at neonatal screening, sTSH and sFT4 levels at the time of diagnosis, and the first dosage of L-T4 (µg/kg/day) used for replacement therapy in the three patient groups.

### 3.2. Characteristics of the Patients during Follow-Up

#### 3.2.1. Serum Hormonal Values

**Figure 2:**
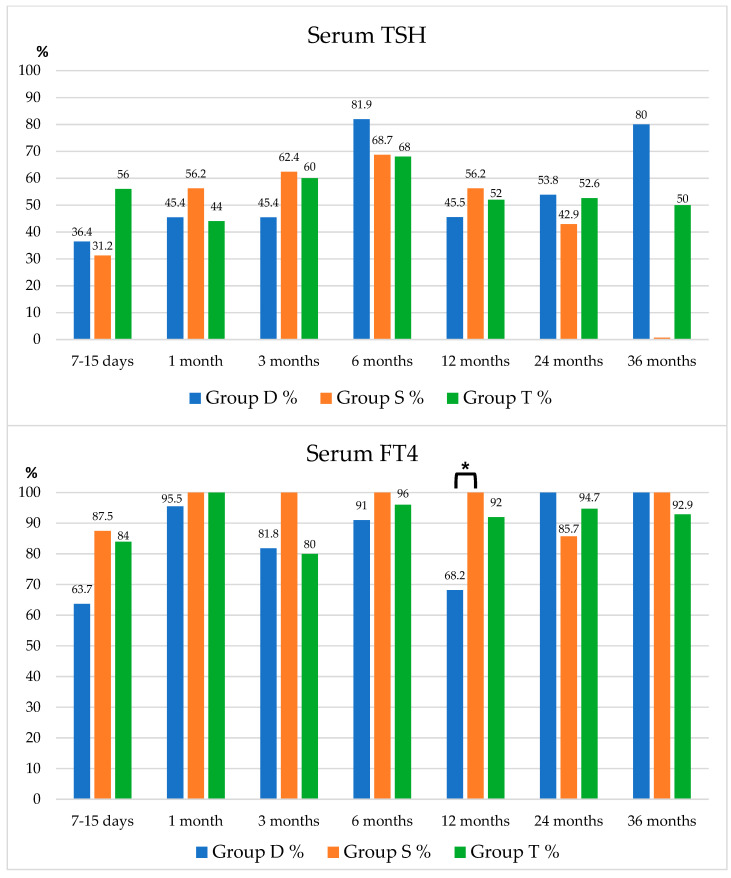
Percentages of patients with sTSH and sFT4 within the normal range (sTSH 0.5–5 µU/mL; sFT4 8.9–22 pg/mL). * *p* = 0.011. In the horizontal axis, the time value is specified, while in the vertical axis, the percentage value is specified.

The three groups showed no significant statistical differences in terms of percentages of sTSH within the normal range, as depicted in Figure 2. The sFT4 levels returned to normal in most patients in all groups during the first month. Additionally, more than 60% of patients in each group already exhibited normal sFT4 levels within 7–15 days (Figure 2). No significant disparities were seen even after 36 months; however, the data is limited by the small sample size.

At 7–15 days from replacement therapy start, the percentage of patients showing sTSH > 5 µU/mL was higher in Group S (68.8% in Group S vs. 28% in Group T, *p* = 0.014; 68.8% in Group S vs. and 50% in Group D, *p* = 0.248). No statistically significant differences in percentages of patients presenting sTSH < 0.5 µU/mL between groups were evidenced.

At 12 months, Group D presented a higher proportion of patients showing reduced sFT4 levels (sFT4 < 8.9 pg/mL) in comparison to Group S (31.8% in Group D vs. 0% in Group S, *p* = 0.011) and Group T (31.8% in Group D vs. 8% in Group T, *p* = 0.038). No further significant differences.

Median sTSH and sFT4 in the three groups at different time-points of the follow-up are reported in Table 3 and Table 4, respectively. At 7–15 days, sTSH levels were significantly greater in Group S, achieving a statistically significant difference only versus Group T.

#### 3.2.2. L-T4 Dosage

**Figure 3:**
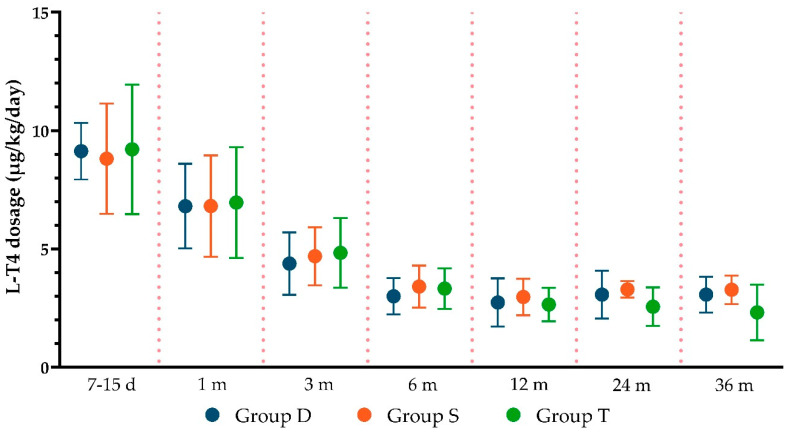
L-T4 mean dosage at each time point of follow-upd = days; m = months).

During follow-up, L-T4 dosage was modified based on thyroid function at blood exams and the patient’s weight. No statistically significant differences between the three groups in L-T4 dosages used were reported (Figure 3).

#### 3.2.3. Frequencies of Treatment Underdose and Overdose

**Figure 4:**
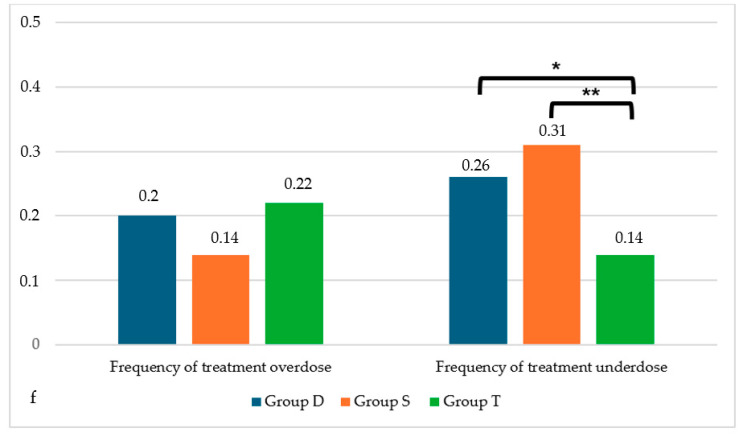
Frequencies of treatment overdose (left) and underdose (right). * *p* = 0.04. ** *p* = 0.016. *f =* in vertical axis frequency of treatment overdose is the number of sTSH < 0.5 µU/mL/total number of sTSH examinations, while underdose is the number of sTSH > 5 µU/mL/total number of sTSH examinations.

Using similar L-T4 dosages, a significant statistical difference was highlighted in frequency of undertreatment (*p* = 0.029): Group D and Group S showed a significantly higher frequency of undertreatment compared with Group T (Group D vs. Group T, *p* = 0.04; Group S vs. Group T, *p* = 0.016), as reported in Figure 4. Non-statistically significant differences during the second and third years.

### 3.3. Anthropometric Parameters

We evaluated height and weight growth, considering the Standard Deviation Score (SDS). No statistically significant differences between groups were found.

Mean weight SDS at 12 months of follow-up was similar in the three groups (Group D: 0.4 ± 1 SDS; Group S: 0.2 ± 0.9 SDS; Group T: −0.03 ± 1 SDS; *p* = 0.325).

**Figure 5:**
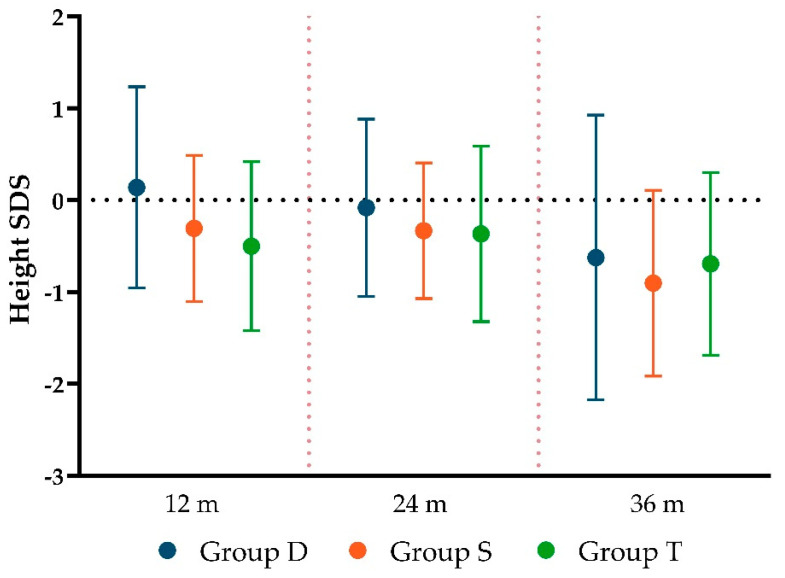
Height SDS during follow-up. SDS: Standard Deviation Score.

The three formulations showed a comparable outcome in height growth (Figure 5), with no differences at 12 months (*p* = 0.072), 24 months (*p* = 0.715), and 36 months (*p* = 0.921). Mean height SDS at 36 months of age was at the lower limit of the normal range (−0.63 ± 1.1 SDS), with no significant differences between groups (*p* = 0.658).

### 3.4. Psychomotor and Neurocognitive Outcome

**Figure 6:**
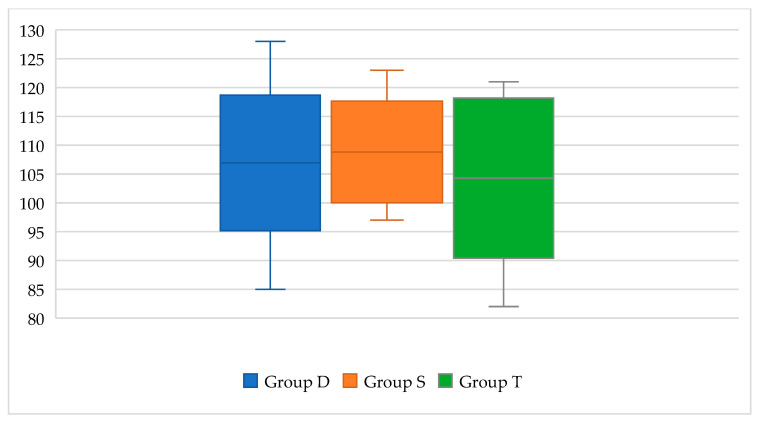
GMDS GDQ at 12 months of age. GMDS: Griffiths Mental Development Scales; GDQ: Global Developmental Quotient.

The neurodevelopmental examination was evaluated at 12 months of age on 35 patients (17 patients in Group D, 11 patients in Group S, and 7 patients in Group T) using GMDS. We observed a similar median GDQ (Group D: 104, range 85–128; Group S: 108, range 97–123; Group T: 110, range 82–121) and GMDS sub-scale scores between the groups (Figure 6 and Table 5). Only one patient, belonging to Group T, presented a GDQ of 82, below the normal range (85–115); this case was a CH due to thyroid ectopy with no apparent further risk factors. Sub-scales scores are reported in Table 5.

At 36 months of age, WPPSI-III confirmed an appropriate and comparable psychomotor outcome in the three groups, though evaluated on a small sample (Group D: 3 patients, Group S: 1 patient, Group T: 4 patients).

## 4. Discussion

Our study observed a comparable efficacy of the three L-T4 formulations in CH treatment. The timing of sFT4 normalization was consistent across all three groups, with most patients achieving normality within 7–15 days from the start of replacement treatment. As previously reported, the normalization of sTSH levels often occurs later than the sFT4 [17]. This was also observed in our study, where only 30–50% of patients had sTSH within the normal range at 7–15 days. However, at this time point, we found a major occurrence of underdosing (higher median sTSH levels and greater percentage of patients with sTSH > 5 µU/mL) in Group S, despite the use of a similar L-T4 dosage, achieving statistical significance only versus Group T.

These findings appear in contrast with the preliminary data of Tuli et al. [18], as well as our previous studies [5,6]. The first examined the liquid formulation in drops and oral solution and found a greater proportion of patients treated with oral solution showing suppressed sTSH at 30 days, compared to those treated with drops. Our prior investigations compared drops and tablets and observed that a higher percentage of individuals treated with drops had suppressed levels of sTSH compared to tablets. In our prior research [5], we hypothesized that liquid formulations in the form of drops would have improved bioavailability and faster absorption. Additionally, there may be a partial loss of the active ingredient when the medication is crushed for administration in children.

To our knowledge, this study is the first to assess all three formulations: oral solution, drops, and tablets. The comparison of our current results with previous studies is challenging to interpret.

However, in our present research, we have noticed that the mean L-T4 doses used were lower (10.1 µg/kg/day ± 2.1 SD) compared to prior studies [5,6], although the first L-T4 dosage was made depending on CH onset severity according to the recommendations of the European Society for Pediatric Endocrinology [1]. Only one kid in Group T was provided the maximal dosage of L-T4 upon diagnosis. This treatment strategy is based on previous research findings, indicating that extended overtreatment may lead to long-term neurodevelopmental side effects [7,8]

The preliminary results described in this study, especially concerning patients treated with oral solution, necessitate further investigations on a larger series of patients. If verified, these findings could suggest a distinct pharmacokinetic profile for the oral solution compared to the drops and induce the use of slightly higher dosages of oral solution at the beginning of replacement therapy. However, it is important to note that the apparent decreased therapeutic efficacy of the oral solution, despite using comparable L-T4 dosages, was only observed in the first 7–15 days after therapy started, and this difference faded in later time points. Furthermore, at 7–15 days of follow-up, sFT4 levels were similar.

At 12 months, we observed a greater proportion of patients in Group D with sFT4 levels below the normal range than in Group S and Group T, despite administering similar dosages of L-T4. This discovery may be attributed to prior clinical experience, leading to more prudent modifications in the L-T4 dose. In this respect, we noted that the average dose of L-T4 was lower at 12 months compared to the other time points. This may indicate the necessity for closer monitoring during this period, when laboratory tests tend to become less concentrated over time due to the unstable L-T4 demand during this rapid growth phase. A well-timed adaptation of L-T4 dosage is crucial to ensure appropriate sFT4 levels for the patient’s age, particularly in patients receiving drops, as they require more precise dose adjustments due to faster absorption. Hence, it might be advantageous to maintain a follow-up interval of 2–3 months till the age of 2 years, rather than one year [1].

During the second and third years of life, we observed an increasing trend of sTSH values, although stable sFT4 levels in all groups. This pattern may be attributed to several variables, including the elongation of monitoring time intervals and decreased promptness in therapy adjustments, or the worse therapeutic compliance due to a kind of “habit” to pathology.

The auxological parameters were consistent across the three groups during the whole follow-up period. At 36 months, the mean height SDS of all groups was at lower limit of the normal range. It is essential to monitor the long-term growth of these individuals up to their final height, particularly considering the potential negative consequences of delayed adjustment of L-T4 treatment observed in our study.

The psychomotor and neurocognitive assessment conducted on the GMDS at 12 months of age revealed a satisfactory DQ. The mean DQ score was similar across all groups and exceeded the average DQ score of age-matched healthy subjects. Sub-scale DQ scores were likewise comparable and fell within the normal range. Neurodevelopmental assessments on the WPPSI-III scale have been conducted on a limited number of patients, due to the small sample size who have completed a 36-month follow-up and the shortage of available data. These results are in agreement with previous studies [5,6,19]. However, to confirm the effectiveness of liquid formulations on psychomotor and neurocognitive outcomes, particularly at 36 months, a more extensive neurodevelopmental follow-up and a larger series of patients are required. This would allow for an assessment of the potential impact of undertreatment, as indicated by this study, as well as an exploration of other factors such as the severity of congenital hypothyroidism, family education, and the ethanol excipient used in the drop formulation.

Liquid formulations (drops and oral solutions) are currently the most used L-T4 formulations in CH treatment. This can be explained by the greater feasibility of administration in infants and children, but also because they allow slight dose adjustment and personalization of therapeutic dosage. Furthermore, the major bioavailability and speed of absorption, as well as explaining the greater risk of overtreatment reported in previous studies [5,6], can be advantageous in the weaning period, in which the introduction of solid foods could compromise the absorption of L-T4 in tablets.

As discussed earlier [6], the inclusion of ethanol excipients in liquid formulations in drops remains a subject of debate. Although the maximum daily L-T4 dosage used at diagnosis (15 µg/kg/die) has an ethanol amount of 36.45 mg/kg, which is four times lower than the danger threshold set by the American Academy of Pediatrics [9,10], long-term potential adverse effects are yet unclear. Few studies have examined the effects of indirect exposition during pregnancy and breastfeeding, reporting that a cumulative daily intake of up to 75 mg of ethanol may be considered safe in long-term follow-up [20,21,22].

Given the shortage of data on direct and extended exposure to ethanol, it is essential to monitor any side effects associated with this excipient. Comparing the long-term efficacy and, above all, safety of drops with an ethanol-free liquid formulation might provide valuable insights. Due to the lack of this excipient and the handling qualities of liquid formulations, the oral solution has the potential to become the most often used formulation in CH therapy.

The primary limitation of this study is the small sample size, especially during the third year of follow-up, which compromises the reliability of the results about hormonal profile and neurodevelopmental evaluation at 36 months of age. In addition, the brief follow-up does not allow for the assessment of the long-term efficacy and safety of L-T4 formulations, nor does it permit the differentiation between transient and permanent CH, which has not yet undergone diagnostic re-evaluation.

## 5. Conclusions

Our preliminary research findings indicate that the L-T4 oral solution is equally effective as drops and tablets in the treatment of CH. This is demonstrated by the lack of statistically significant differences between the group treated with an oral solution and the other two groups in laboratory assessments of thyroid functioning throughout follow-up, except at 7–15 days after the treatment start. At this time, there was a higher frequency of undertreatment in the group receiving the oral solution: a greater percentage of patients with sTSH > 5 µU/mL and higher sTSH levels were observed in the oral solution group compared to groups treated with drops and tablets. Nevertheless, these variations were not linked to any disparities in sFT4 levels. The clinical findings also demonstrate comparable effectiveness since there were no observed differences in growth and neurodevelopmental outcomes. Our results need to be validated by prospective studies involving larger patient series and longer observation periods.

## Figures and Tables

**Table 1:** Population characteristics at diagnosis. N: number; M: male; F: female; SDS: standard deviation score; SD: standard deviation.

		Group D	Group S	Group T
Subjects Number		22	16	25
**Sex**, N (%)	M	9 (40.9%)	7 (43.7%)	7 (28%)
	F	13 (59.1%)	9 (56.3%)	19 (72%)
**Familiarity for thyroid** **disease**, N (%)		9 (40.9%)	9 (56.3%)	15 (60%)
**Comorbidities**, N (%)		1 (4.5%)	0	2 (8%)
**Gestational Age**, median (min-max)		40.1 (36–41.6)	39.5 (37.4–42)	40 (36–41.7)
**Birth weight SDS**, mean ± SD		−0.4 ± 0.9	0.03 ± 0.9	−0.5 ± 0.8
**Birth length SDS**, mean ± SD		−0.1 ± 0.9	0.07 ± 1	−0.25 ± 1.1
**Dysgenesis**, N (%)		16 (72.7%)	12 (75%)	12 (48%)

**Table 2:** Laboratory characteristics at diagnosis and L-T4 dosage at the beginning of replacement therapy. DBS: dried blood spot; sTSH: serum thyroid stimulating hormone; sFT4: serum free thyroxine; SD: standard deviation.

	Group D	Group S	Group T
**DBS TSH value** median (min–max), µU/mL	79 (9–200)	117.2 (9–200)	26.4 (9–200)
**sTSH value** median (min–max), µU/mL	262.1 (29.1–467)	303.9 (21.2–467)	78.5 (15.5–493)
**sFT4 value** median (min–max), pg/mL	6.3 (2.5–12.3)	5.1 (2.7–14)	8.4 (2.5–19.5)
**L-T4 dosage** mean ± SD, µg/kg/day	10.2 ± 1.5	9.5 ± 2.4	10.4 ± 2.5

**Table 3:** Median serum TSH levels during follow-up. * Group S vs. Group T (*p* = 0.031).

Time Point	Group D		Group S		Group T	
	sTSH, µU/mL (Min–Max)	N° pts	sTSH, µU/mL (Min–Max)	N° pts	sTSH, µU/mL (Min–Max)	N° pts
7–15 days	5 (0.2–285.6)	22	13.1 (1.2–157.8) *	16	3.1 (0.2–317.6) *	25
1 month	0.9 (0.03–181.7)	22	3.6 (0.2–20)	16	0.8 (0.03–93.1)	25
3 months	0.8 (0.01–9.3)	22	1.3 (0.01–5.1)	16	0.8 (0.03–6.7)	25
6 months	1.3 (0.2–15.4)	22	1.5 (0.01–8.8)	16	2.4 (0.1–25.3)	25
12 months	5.1 (0.1–43.5)	22	4.2 (0.6–11.7)	16	2.4 (0.1–59)	25
24 months	2.6 (0.3–8.8)	13	6 (1.6–14.2)	7	4.2 (0.5–14.5)	19
36 months	3.4 (2.3–5.6)	5	13.3 (5.5–21.1)	2	4.7 (0.8–22.7)	14

**Table 4:** Median serum FT4 levels during follow-up.

Time Point	Group D		Group S		Group T	
	sFT4, pg/mL (Min–Max)	N° pts	sFT4, pg/mL (Min–Max)	N° pts	sFT4, pg/mL (Min–Max)	N° pts
7–15 days	18.1 (7.4–28.2)	22	16.1 (10.3–29)	16	15.4 (8.4–40.6)	25
1 month	14.5 (9.6–24.3)	22	13.9 (9.5–19.2)	16	14 (10.9–20.8)	25
3 months	12.7 (7.6–23)	22	12.9 (9.4–15.3)	16	12.3 (3.9–22.4)	25
6 months	10.5 (7.9–14.1)	22	11.3 (9.4–13)	16	11.2 (8.7–16.8)	25
12 months	10.2 (7–15.2)	22	10.6 (9.1–12.6)	16	10.5 (8.3–16.3)	25
24 months	11.3 (9.5–14)	13	11 (1.6–14.7)	7	10.7 (7.6–17.8)	19
36 months	13.6 (11.2–16.2)	5	12.6 (10.9–14.2)	2	10.9 (1.4–16.1)	14

**Table 5:** GMDS sub-scales DQ at 12 months of age. GMDS: Griffiths Mental Development Scales; DQ: Developmental Quotient.

GMDS Sub-Scales	Group D	Group S	Group T
**Locomotor**, median (min–max)	103 (61–112)	95 (66–108)	87 (82–113)
**Personal-social**, median (min–max)	100 (79–133)	102 (81–114)	96 (80–113)
**Hearing and language**, median (min–max)	110 (81–133)	106 (98–135)	118 (76–139)
**Eye and hand coordination**, median (min–max)	112 (95–140)	120 (77–131)	117 (74–123)
**Performance**, median (min–max)	111 (90–131)	115 (105–143)	108 (98–123)

## Data Availability

The original contributions presented in the study are included in the article, further inquiries can be directed to the corresponding author.
